# Improving PrEP Implementation Through Multilevel Interventions: A Synthesis of the Literature

**DOI:** 10.1007/s10461-018-2184-4

**Published:** 2018-06-05

**Authors:** Rogério M. Pinto, Kathryn R. Berringer, Rita Melendez, Okeoma Mmeje

**Affiliations:** 1 School of Social Work, University of Michigan, Ann Arbor, MI USA; 2 Sociology and Sexuality Studies, San Francisco State University, San Francisco, CA USA; 3 Department of Obstetrics and Gynecology, University of Michigan Medical School, Ann Arbor, MI USA; 4 University of Michigan School of Social Work, Room 2850, 1080 South University, Ann Arbor, MI 48109 USA

**Keywords:** PrEP implementation, PrEP integrative review, HIV prevention, Continuum of care

## Abstract

There are many challenges to accessing PrEP and thus low uptake in the United States. This review (2007–2017) of PrEP implementation identified barriers to PrEP and interventions to match those barriers. The final set of articles (n = 47) included content on cognitive aspects of HIV service providers and individuals at risk for infection, reviews, and case studies. Cognitive barriers and interventions regarding patients and providers included knowledge, attitudes, and beliefs about PrEP. The “purview paradox” was identified as a key barrier—HIV specialists often do not see HIV-negative patients, while primary care physicians, who often see uninfected patients, are not trained to provide PrEP. Healthcare systems barriers included lack of communication about, funding for, and access to PrEP. The intersection between PrEP-stigma, HIV-stigma, transphobia, homophobia, and disparities across gender, racial, and ethnic groups were identified; but few interventions addressed these barriers. We recommend multilevel interventions targeting barriers at multiple socioecological domains.

## Resumen

Existen muchos desafíos para acceder a PrEP y, por lo tanto, poca aceptación en los Estados Unidos. Esta revisión (2007-17) de la implementación de PrEP identificó las barreras a la PrEP y las intervenciones para hacer coincidir esas barreras. El conjunto final de artículos (n = 47) incluyó contenido sobre los aspectos cognitivos de los proveedores de servicios de VIH y las personas en riesgo de infección, revisiones y estudios de casos. Las barreras cognitivas y las intervenciones con respecto a los pacientes y proveedores incluyeron el conocimiento, las actitudes y las creencias sobre la PrEP. La “purview paradox” se identificó como una barrera clave: los especialistas en VIH a menudo no ven pacientes VIH negativos, mientras que los médicos de atención primaria, que a menudo ven pacientes no infectados, no están capacitados para proporcionar PrEP. Las barreras de los sistemas de salud incluyen la falta de comunicación, financiación y acceso a la PrEP. Se identificó la intersección entre el estigma de la PrEP, el estigma del VIH, la transfobia, la homofobia y las disparidades entre los grupos de género, raciales y étnicos; pero pocas intervenciones abordaron estas barreras. Recomendamos intervenciones multinivel dirigidas a las barreras en múltiples dominios socioecológicos.

## Introduction

In 2011–2012, the United States Centers for Disease Control and Prevention (CDC) launched the high-impact HIV prevention (HIP) approach to respond to research showing that antiretroviral therapy (ART) reduces HIV transmission by lowering viral load in the bloodstream [1]. In 2012, the use of ART emerged as the dominant strategy for HIV treatment and prevention [2]—research predicted reduction of sexual transmission in HIV-serodiscordant couples by more than 96% [3]. High-impact interventions—HIV testing, linkage to care, and HIV viral suppression with ART—constitute key steps of the HIV Continuum of Care, recommended by the World Health Organization [4, 5]. HIP promotes HIV pre-exposure prophylaxis (PrEP). PrEP has been traditionally considered as once daily oral dosing of ART prescribed to individuals at risk for HIV infection. The Food and Drug Administration (FDA) approved Truvada™ [Emtricitabine/Tenofovir Disoproxil Fumarate (TDF/FTC)] in 2012 as a PrEP strategy that reduced the risk of HIV acquisition by 73% among adult men who have sex with men (MSM) and transgender women who took it 90% of the time [6]; with greater efficacy (up to 99%) for individuals with higher rates of adherence and increased concentrations among serodiscordant heterosexual couples [7, 8].

Herein, the steps patients and providers must take to follow policies governing access to PrEP and to navigate healthcare systems will be referred to as “PrEP implementation.” PrEP implementation may appear to be an easy and effective way to stop HIV transmission; however, there are many challenges to accessing and adhering to PrEP, as reflected in low levels of PrEP uptake in the US [9, 10]. Concern over rates of adherence and retention have been reported in PrEP care in clinical trials and “real world” PrEP demonstration projects [11, 12]. Racial and gender disparities have also been identified, including disproportionately low PrEP uptake among Black MSM [13]. Research regarding low access, uptake, and adherence to PrEP in the US has focused mostly on breakdowns in the healthcare systems implementing PrEP, lack of provider awareness and willingness to prescribe PrEP [9, 14], and unfavorable patient and community attitudes about PrEP [15, 16]. Our aim therefore is to comprehensively review this literature, focusing on how barriers to PrEP uptake might affect both individual actors and healthcare systems.

Barriers to PrEP implementation occur across gender, racial, and ethnic groups. Various interventions have been proposed to solve this public health problem, including those targeting different domains of prevention and care—patients, providers, and healthcare systems. Nonetheless, proposed interventions to improve PrEP implementation may vary across the fields of medicine, nursing, social work, public health, and the social sciences. Therefore, this review included papers in all these disciplines and sought to identify barriers to PrEP and the interventions available that specifically matched those barriers. We focused specifically on PrEP implementation in the US and on implementation issues faced by at-risk individuals and HIV service providers, the agency settings in which services are offered, and the policies that guide HIV service provision. “HIV service providers” in this context refer to counselors, educators, case managers and others who provide HIV testing, linkage to care, and other services, as well as HIV-care providers. Our review aims to improve HIV-prevention strategies nationwide by demonstrating how to confront identified barriers with interventions that might improve access, uptake, and adherence to PrEP.

## Methods

### Literature Review Conceptual Approach

**Fig. 1:**
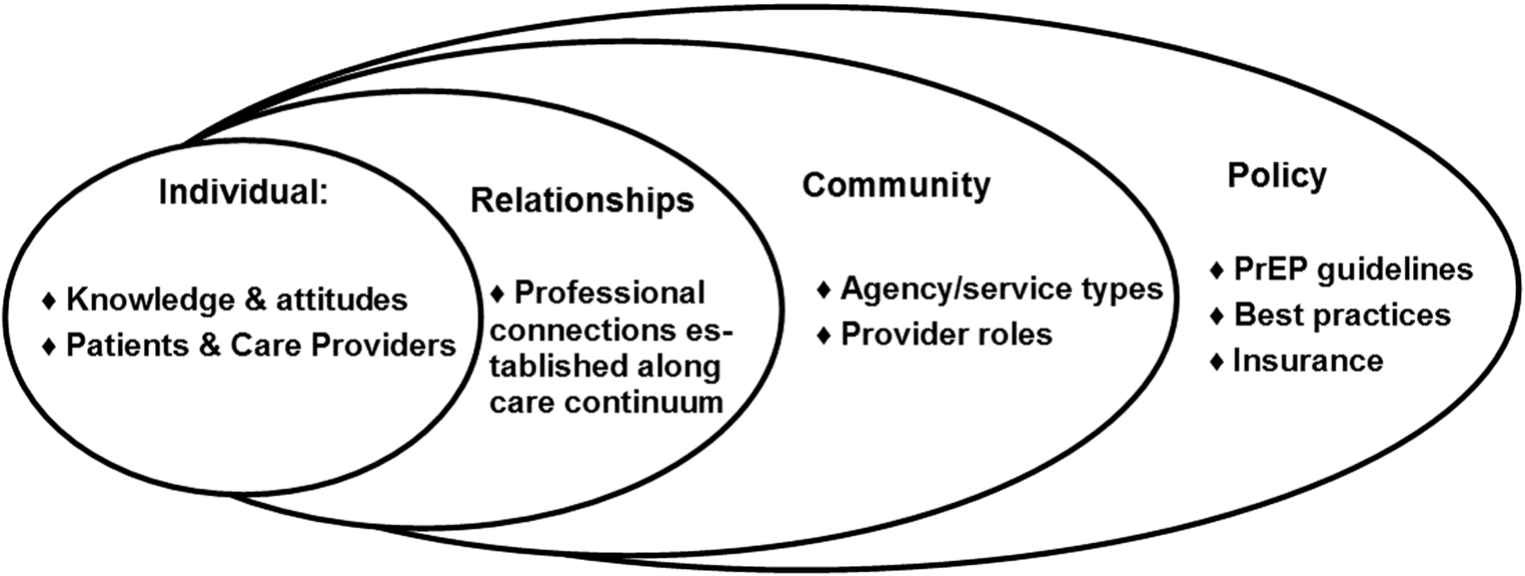
Conceptual approach: socioecological barriers to PrEP implementation

The extant literature consistently suggests that to improve PrEP implementation, key barriers need to be overcome and interventions need to be developed at all levels (clients/patients, HIP service providers, healthcare systems, and policy) [17–19]. Therefore, our review follows a socioecological perspective [20] suggesting that barriers to PrEP implementation fall within four domains. The Individual and Relationships Domains represent patients and care providers, as well as the professional connections they establish along the HIV Continuum of Care that may hinder or facilitate PrEP implementation; barriers in this domain involve knowledge of, attitudes about, and burdens regarding PrEP implementation. The Community and Policy Domains represent policies governing HIV-prevention efforts, including PrEP implementation across healthcare systems and agency settings in communities where at-risk individuals access PrEP; barriers in this domain include structural factors in healthcare systems, and governmental and health-organization guidelines that might hinder PrEP implementation. The socioecological perspective, summarized in Fig. 1 (modified from Mugavero et al. 2013), is consistent with ecological models in public health and epidemiology, from Bronfenbrenner’s ecological systems theory to more-recent theories in social epidemiology [21]. This approach focuses on individuals within larger social environments (patients) and institutional environments (care providers); significantly, it distinguishes between interventions that target individuals and their environmental and structural contexts. Our approach to PrEP implementation acknowledges that both individuals and their healthcare providers are embedded within larger healthcare systems governed by multiple policies [22, 23].

### Integrative Review Model

We adopted an integrative review model to provide a more comprehensive understanding of PrEP implementation in various domains of reference [24]. Our review focuses on a period (January 2007 to June 2017) that included the emergence of the concept of the HIV Continuum of Care and the high-impact prevention (HIP) approach, followed by large-scale clinical trials (e.g., the iPrEx study) [6, 25, 26], and the subsequent approval by the FDA (in July 2012) of the provision of PrEP in service settings [27].

### Literature Search Terms

We used combinations of search terms in Articles*Plus*, a comprehensive database of peer-reviewed clinical and academic journals in medicine, public health, social work, nursing, pharmacy, and law, hosted by the University of Michigan Library. Our combination of search terms, including truncation operators (*), was as follows:

**Subject Terms:** (HIV OR HIV/AIDS OR AIDS) AND

**Title:** (PrEP OR “Pre-Exposure Prophylaxis”) OR [(antiretroviral* OR pharmaceutical*) AND prevent*)] AND

**All Fields:** [(worker* OR practitioner* OR provider*) AND (linkage* OR linking OR referral* OR implementation OR uptake)]

### Inclusion and Exclusion Criteria

We included peer-reviewed papers presenting research on PrEP implementation, PrEP in the US, HIV service workers, practitioners, medical or social-service providers, and service agencies. We excluded papers that exclusively addressed the attitudes and beliefs of individuals targeted by HIV-prevention programs; however, we did include studies focused on the attitudes and beliefs of potential PrEP patients specifically related to the effective implementation of PrEP. We also included studies focused on attitudes and beliefs of HIV-prevention providers. We restricted this review to research in the US because of the unique historical response to HIV/AIDS in the US and the particular attributes of its healthcare system, and to yield results applicable to PrEP implementation in the US.

### Procedures for Article Selection

**Fig. 2:**
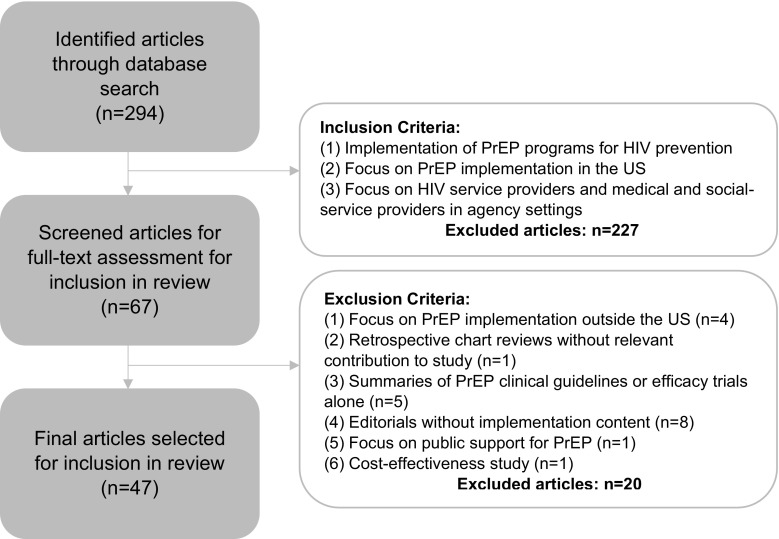
Summary of article selection and inclusion and exclusion criteria

Figure 2 summarizes our procedures for article selection. Our initial search yielded 294 articles. Following our inclusion criteria, we first read titles and abstracts and screened out 227 papers that did not match our criteria. For example, in this screening we excluded articles focusing on PrEP implementation outside the US and papers exclusively addressing attitudes and beliefs of individuals (e.g., PrEP acceptability studies)—yielding 67 articles, which were fully assessed. As we read and discussed the articles, we screened out another 20 whose contributions lacked relevance to the study of PrEP implementation (e.g., editorials on the promise of PrEP alone, studies of public support, and cost-effectiveness studies), bringing our final sample to 47 articles. To organize and manage our library, we created an Excel spreadsheet to record key information about each publication: title, authors, journal, publication date, journal type, methods and methodology, and a summary of findings.

### Analysis

Our analysis focused on identifying barriers to PrEP and the interventions aligned with those barriers. To enhance the rigor of our analysis, we adopted the following techniques: purposive sampling; grounded theory; and multidisciplinary collaborative interpretation [28].

#### Purposive Sampling

We borrow the term “purposive sampling” to describe the procedures we used (described above) to select the articles for this integrative review—specific search terms, inclusion and exclusion selection criteria, and procedures for article selection.

#### Grounded Theory

Our analysis reflects a modified version of grounded theory [29] in which how we selected the final set of articles for analysis, as well as how we read/interpreted the articles, was based on the content we found and grounded in our experiences as both HIV/AIDS researchers and practitioners in community settings offering HIV-related services. We concurred on definitions, recurring terms, and barriers and solutions to PrEP implementation before beginning analysis. We also used a conceptual framework to guide how we identified and aligned barriers with interventions in the selected articles.

#### Multidisciplinary Collaborative Interpretation

The examination of the 47 selected articles included a full-text reading of each paper. The list of articles was ordered by relevance to our search terms and this order was maintained throughout the review. Articles were discussed by the first two authors, with expertise in social work and anthropology, grounded in the conceptual foundation above. Given the close connections across barriers and interventions regarding patients and providers, we combined them under the “Individual and Relationships Domains.” Since healthcare systems operate within communities and are concurrently influenced by myriad policies, we combined healthcare-system barriers and interventions under “Community and Policy Domains.” To address researcher bias, we used rigorous procedures (described above) to select articles for the review. The first two authors held seven weekly 60-min discussions to finalize the list of barriers and matching interventions and came to 100% agreement. This list was presented to the third (sociologist) and fourth (medical doctor) authors for further interpretation. Based on our shared interpretation and judgment, we organized a pragmatic list (Table 1).

**Table 1:** Summary of barriers to, and interventions to improve, PrEP implementation

Conceptual domain and intervention level	Barriers to PrEP implementation	Interventions matching specific barriers
Individual and Relationships Domains: Provider Level	*Knowledge* Lack of training in PrEP provision Disagreement/uncertainty about appropriate PrEP patients Concerns/uncertainty about insurance coverage for PrEP *Attitudes and beliefs* Biases against patients’ race and sexual behaviors Concerns about PrEP efficacy, toxicity, and resistance Concerns about patients’ disinhibition and risk compensation leading to lack of adherence/compliance	*Knowledge* Improved education of potential PrEP providers Development of trainings and interventions to assist providers in identifying appropriate PrEP candidates *Attitudes and beliefs* Development and delivery of trainings to increase provider “cultural competency,” including trans- and gender-affirming care Interventions to identify and disrupt provider-held stereotypes about potential PrEP users
Individual and Relationships Domains: Patient Level	*Knowledge* Low awareness of PrEP and low demand for PrEP *Attitudes and beliefs* Side effects; effectiveness; toxicities; interaction with feminizing hormones Managing multiple health concerns and PrEP side effects Prioritization of care for current conditions (e.g., pain or stress) above HIV prevention Prioritization of gender-affirming feminizing hormone therapy Distrust of medical system: structural racism, transphobia, and negative experiences Competing priorities during periods of substance use Diminished concern for prevention with intimate partners Concerns about HIV-reporting systems, including potential insurance implications of a positive HIV result Unwillingness to discuss PrEP with primary care providers	*Knowledge* Increased education and counseling to increase PrEP knowledge *Attitudes and beliefs* Development of supportive behavioral interventions (e.g., risk-reduction, medication-adherence, and retention counseling) Assistance in navigating the healthcare system, including accessing health insurance and co-pay assistance Referrals of patients with mental-health, substance-use, or “social” issues (e.g., housing insecurity) to social workers or community resources Side-effect monitoring
Community and Policy Domains: Healthcare-System Level	*Communication and awareness* Lack of effective messaging about PrEP Lack of communication between healthcare providers and community-based organizations *Funding* Limited health budgets to sustain PrEP programs Lack of insurance coverage and financial-assistance programs *Capacity & access* Lack of focus on “nonprescribing service providers” Purview paradox: neither HIV specialists nor PCPs consider PrEP implementation within their clinical domain Lack of training, referral systems, or established reimbursement levels for care and drugs Legal constraints to providing PrEP for youth, including mandates to involve parental figures in working with minors Lack of access to care: inadequate transportation; inflexible work schedules; inconvenient locations dispensing PrEP Time constraints on medical appointments Lack of medical insurance and limited insurance networks Lack of patient confidence and perseverance to access care *Pharmaceutical barriers* Particular constraints of Truvada™ as PrEP (e.g., daily dosing schedule, side effects) *Population-specific barriers and stigma* Lack of gender-affirming healthcare for transgender women Lack of trans-inclusive marketing of PrEP Low prioritization of PrEP for people who inject drugs Stigma associated with PrEP use and accessing HIV services The intersection of HIV-stigma with transphobia and homophobia	*Communication and awareness* Community-engagement and community-mobilization strategies Systems to improve interagency/interprofessional collaboration *Funding* General advocacy for expanded health insurance Funding for medication costs, adherence counseling/monitoring, and support services; referral to medication-assistance programs *Capacity and access* Expanded PrEP-delivery systems, staff, time, space, expertise Engagement of generalist PCPs in PrEP provision for scale-up (addressing the purview paradox) Expanded/diversified settings providing PrEP (e.g., private practices, mental-health clinics, ERs) and integration of PrEP into primary care Expanded education, screening, referrals to PrEP services Improved methods to identify appropriate PrEP candidates Specific guidelines from “normative bodies” (e.g., CDC, APA) Partnerships between medical and social-service providers Development of systems to monitor and evaluate PrEP use Cross-training of staff (e.g., educators, pharmacists, nurses) Improvements in pharmacists’ PrEP education *Pharmaceutical barriers* Advancing new PrEP technologies: innovative pharmacologic chemoprophylactic approaches (e.g., on-demand PrEP dosing, injectable, microbicides, rings, films) Pharmacokinetic studies of potential drug–drug interactions, particularly in oral PrEP medications and feminizing hormones *Population-specific barriers and stigma* Disaggregating transgender women from MSM in research and clinical practice and developing trans-inclusive research strategies Improving access to trans-competent PrEP providers Integrating PrEP care with contraceptive services Focusing resources on vulnerable communities Expanded “youth-friendly” health services, including augmented PrEP visit schedules, adherence clubs and social-support groups

## Results

The final set of articles (n = 47) included four broad categories of papers: primary data on cognitive variables (e.g., perspectives, beliefs, and concerns) of HIV-prevention providers (n = 18) [9, 14, 30–45]; primary data on cognitive variables and perspectives of individuals considered at risk for HIV infection (n = 9) [46–54]; reviews of current literature on PrEP implementation (n = 16) [10, 11, 17, 19, 55–66]; and case studies of PrEP demonstration and implementation projects (n = 4) [12, 54, 67, 68]. From the text of these articles, we extracted and recorded key barriers to implementation and the interventions proposed to address them. Then we mapped out the barriers. Among patients and providers, we identified cognitive barriers and interventions regarding their knowledge, attitudes, and beliefs about PrEP. Barriers involving healthcare systems included communication and awareness about PrEP, lack of funding and/or insurance, and capacity and access. We also identified pharmaceutical- and population-specific barriers. Below we provide an account of these barriers and the interventions that might address them, thus improving PrEP implementation. Table 1 provides a summary of these findings.

### Barriers to and Interventions with Potential to Improve PrEP Implementation

#### Individual and Relationships Domains—Provider Level

Eighteen articles focused on primary care physicians, HIV and infectious-disease specialists, pharmacists, and nurse practitioners—including analyses of focus groups, interviews, and surveys. None of the papers included data from social-service providers, though most of them mentioned the need for the expansion of referrals to mental-health and other support services, care coordination, and peer-based groups, all of which might improve PrEP implementation [10, 30, 32, 46, 57, 66]. Providers described concerns and solutions across socioecological levels. For instance, proposed system-level solutions included engaging generalist physicians in PrEP provision [31], community education campaigns [32], and increased funding for counseling and social support services [32].

Significantly, many of these papers noted the “purview paradox”—the idea that the providers who are best trained and most willing to prescribe PrEP (i.e., HIV specialists) often do not see HIV-negative patients who would benefit from PrEP, while physicians who regularly care for HIV-negative patients (i.e., primary care physicians) are often not trained to provide PrEP [14, 56]. Other barriers included providers’ lack of knowledge, negative attitudes toward PrEP, lack of training in PrEP provision, disagreements about who might be appropriate candidates for PrEP use, and concerns about insurance coverage for PrEP. The solutions proposed to address knowledge gaps included trainings and interventions to assist providers in identifying appropriate PrEP candidates. We also found that prejudicial beliefs (e.g., assessments of the likelihood of risk behavior based on race) [38], concerns about the efficacy (or “real world” efficacy) of PrEP, toxicities, and future resistance, and about patients’ behavior (e.g., sexual risk and lack of adherence) were often reported as substantial barriers to PrEP implementation [56].

#### Individual and Relationships Domains—Patient Level

Nine articles reported on primary data about potential PrEP patients’ attitudes, beliefs, and experiences. Most included qualitative interviews and results of focus groups with community members. We considered data from provider perspectives on patient-level barriers and solutions—for example, perceived barriers regarding increased risk behaviors associated with PrEP use. Barriers cited included patients’ lack of knowledge and low demand for PrEP, as well as socioeconomic (e.g., stigma and difficult access to transportation) and medical burdens (e.g., side effects of PrEP) that complicate PrEP uptake. Perceived barriers, some not confirmed by strong evidence, included concerns about effectiveness [57], toxicities [11, 14, 32, 69], and interactions with gender-affirming hormones among transgender women [56, 62]. Research cited the higher priority given to care for current conditions, both medical and psychosocial, and gender-affirming hormone therapy than to HIP [46, 48, 49]. The “seasonal” nature of sexual risk trajectories was also reported as a barrier to PrEP [68]. Distrust of the medical system based on historical legacies of structural racism, transphobia, and other forms of discrimination was reported as a significant barrier to PrEP access [10, 62].

The review also revealed a diminished concern about HIP when patients are in intimate partnerships and/or using substances [46, 49], an unwillingness to discuss PrEP with primary care providers, and challenges managing multiple health concerns, in addition to potential side effects from PrEP. While many of these barriers focused on the patient, we found that proposed interventions often necessitated system-level interventions (e.g., expanded access to and capacity for PrEP and targeted interventions to address population-specific barriers to PrEP). Individual-level solutions proposed included targeting knowledge and awareness, attitudes, beliefs, and burdens; focusing on increased education and counseling; and offering supportive behavioral interventions such as risk reduction, medication adherence, and retention counseling [12, 17, 50, 56]. Interventions were proposed to help patients navigate healthcare systems and improve the frequency of referrals to mental health, substance abuse, and other supportive services.

#### Community and Policy Domains—Healthcare-System Level

Twenty studies consisted of broad reviews of existing PrEP literature or reviews of large-scale PrEP demonstration or implementation projects. These studies, as well as those focused on provider and potential-patient perspectives, addressed myriad system-level barriers to PrEP implementation and proposed system-level solutions. We organized the system-level barriers into five categories: problems with communication and awareness; lack of funding and/or insurance; lack of capacity and access; pharmaceutical barriers; and population-specific issues and stigma.

##### Problems with Communication and Awareness

Our review revealed a lack of effective messaging about PrEP and communication between healthcare providers and community-based organizations [52]. Proposed solutions in this domain included community engagement and mobilization strategies [62] as well as systems to improve interagency and interprofessional collaboration.

##### Funding and/or Insurance Barriers

Lack of funding is the most consistently cited system-level barrier, including limited health budgets to sustain PrEP programs and lack of insurance coverage [9, 46, 47, 54, 56]. The latter has been framed as both a systems-level barrier to access and care, with studies showing that patients without access to insurance are less likely to successfully obtain PrEP [54]; and as a provider-level barrier, with insurance barriers affecting providers’ attitudes and behaviors about prescribing PrEP [9]. The cost of PrEP is covered by many health insurance plans [70]. Gilead Advancing Access^®^ program, a commercial medication assistance program, provides free PrEP to eligible HIV-negative adults in the US with limited income and no insurance covering PrEP [71]. However, individuals enrolled in government programs (e.g., Medicare Part D, Medicaid, TRICARE, or VA) are not eligible for this program. Adolescents under 18-years-old and young people covered by their parents’ insurance, and who may wish to seek PrEP independently to avoid disclosure through their parents’ Explanation of Benefits, are also excluded from this program [65, 66, 72]. Private insurers’ policies concerning medications, including PrEP, are insurance-specific and thus outside the scope of this review. However, it is important to mention that insurers have enacted policies that may exacerbate existing barriers to PrEP implementation, such as prior authorization paperwork requirements, and strict requirements regarding completion of test results prior to authorizations and prescription renewals. In addition to suggesting help for patients in navigating healthcare systems to access insurance and co-pay assistance programs, articles proposed general advocacy for expanded health insurance [46], coverage of medication costs, PrEP adherence counseling, and support services [56].

##### Capacity and Access

Barriers included a lack of focus on non-prescribing providers [10]; the purview paradox; lack of referral systems, and lack of training on, for example, when to initiate PrEP; legal constraints to providing PrEP for youth [65]; lack of access to care caused by inadequate transportation, inflexible work schedules, time constraints during medical appointments [56], and inconvenience of locations dispensing PrEP; and lack of medical insurance. Solutions to these barriers included expanded space, time, and expertise for PrEP-delivery systems [67]; engagement of generalist PCPs in PrEP provision (to address the purview paradox); diversification of settings providing PrEP (e.g., mental-health clinics and criminal-justice settings) [46]; integration of PrEP into primary care; education, screening, and referrals to PrEP; improved methods to identify appropriate PrEP candidates [56]; stronger guidelines and policies for providers [34]; partnerships between medical and social-service providers; cross-training of staffers (e.g., social workers, educators, pharmacists, and nurses); leadership support of increased staff time to address financial barriers [12]; and improving pharmacists’ PrEP education [45].

##### Pharmaceutical Barriers

We identified barriers specific to Truvada™ and its oral daily dosing schedule and potential side effects. Proposed solutions included advancing new PrEP technologies, such as pursuing innovative pharmacologic chemoprophylactic approaches (e.g., on-demand PrEP dosing, injectables, microbicides, rings, and films), and pharmacokinetic studies of potential drug–drug interactions, particularly involving those with feminizing hormones [11, 62].

##### Population-Specific Issues and Stigma

Several papers focused on transgender women [49, 53, 62], Black and Latina women [48], Black and Latino MSM [51], adolescents [65, 66], men who engage in street-based sex work [46], heterosexual couples [69], and people who inject drugs [43, 63]. These papers point to stigma associated with PrEP use and the intersection of HIV-stigma with transphobia and homophobia [48, 49]. Despite the number of articles that identify stigma as a barrier to PrEP, few interventions were proposed that would directly address the effects of stigma.

##### Transgender Women

Barriers specific to transgender women included non-inclusive marketing of PrEP; perceived interactions with feminizing hormones and prioritization of hormone care; managing multiple medical appointments and medications; mistrust arising from transphobia in the medical system; and life instabilities and substance use. Proposed gender-affirming healthcare initiatives included prioritizing hormones and gender-affirming medical care, exclusively using patients’ preferred names and pronouns, and creating safe spaces for trans clients [62]. Studies also proposed pharmacokinetic studies of potential drug–drug interactions between oral PrEP medications and gender-affirming hormones in transgender women [11, 62]. Sevelius et al. [49] argue that current deficits in the provision of gender-affirming care for transgender women are connected to the conflation of transgender women with MSM, which serves to conceal transgender women’s unique social and behavioral vulnerabilities.

##### Cisgender Black Women and Latinas

Like transgender women, cisgender Black women and Latinas face particular barriers to engaging with messages often designed for MSM [48]. PrEP implementation among women may be helped by addressing the burden of frequent medical visits; the stigma associated with accessing HIV services; and the burden of pill-taking, including concerns about adding to an existing pill burden. Increasing the availability of PrEP in settings where women receive services may also improve PrEP uptake, for example by integrating PrEP care with provision of contraceptives and screening for sexually transmitted infections [48].

##### Black and Latino Men

Barriers for Black and Latino men include decreased access to private health insurance and more access through public clinics, as well as frequently endorsed stigma-related concerns about PrEP [47, 51]. Healthcare is a problematic area for MSM of color, who are more likely than other men to view talking about their sex lives to their doctors as a barrier to PrEP [51]. Moreover, research included in this review indicates that medical providers in training exhibit prejudicial assessments of Black patients based on stereotypes about risk compensation (e.g., increased condomless sex associated with PrEP use) [38]. While this study was limited to current medical students, exploratory research surveying medical providers (primarily HIV specialists) suggests that providers’ likelihood to prescribe PrEP varies widely across patient groups, making the potential consequences of prejudicial assessments particularly troubling [73]. These barriers underscore the limitations and potentially severe consequences of considering seemingly individual-level interventions (such as provider knowledge or individual behavior) in isolation from larger systemic factors, such as structural racism.

## Discussion

The purpose of this integrative review was to identify barriers to PrEP implementation and interventions to improve it. The 47 reviewed articles reported barriers at all four domains of the conceptual framework. But these barriers rarely exist in isolation, and proposed interventions are not always aligned to specific barriers. For instance, while a number of papers (n = 18) focused exclusively on the perspectives, knowledge, and concerns of providers, these papers rarely offered solutions to overcome barriers related to providers; instead, they offered solutions focused on targeting the behavior of individual patients, such as interventions to improve patient adherence [57] or evidence-based interventions to reduce risk compensation [10]. Moreover, frequently cited barriers to PrEP implementation cut across all three levels, as in the case of the purview paradox [14, 56], and also in the case of structural barriers, such as patient distrust of the medical system based on historical legacies of structural racism and of transphobia [10, 62]. Grounded in our understanding of this literature, we provide a comprehensive picture of how potential changes to PrEP implementation can be mapped onto specific barriers identified in the extant literature. In so doing, we are filling a research gap in the literature.

Given the interconnected nature of the barriers identified, we recommend the adoption of a dynamic social-systems model, as developed by Latkin and colleagues, for PrEP implementation in which individual, dyad, and structural factors are viewed as elements of a complex system in which none functions in isolation (p. S233) [74]. We also suggest (below) specific targets of interventions based on Nunn et al.’s nine-step PrEP care continuum, analogous to the HIV Continuum of Care, as a model for PrEP implementation—identifying individuals at high risk, increasing individual HIV-risk awareness, enhancing PrEP awareness, facilitating PrEP access, linking to PrEP care, prescribing PrEP, initiating PrEP, adhering to PrEP, and retaining individuals in PrEP care [75]. Both these models suggest multilevel interventions to achieve effective PrEP implementation. Multilevel interventions would integrate biomedical, behavioral, and structural or systemic components [17]. Just as patients, providers, and systems do not operate in isolation, proposed interventions cannot be considered to perform isolated functions (e.g., biomedical, behavioral, or structural).

“PrEP navigation”—auxiliary, non-prescribing providers whose role is to assist people in overcoming structural barriers to care [20] is an intervention whose potential to address barriers in different socioecological domains has been acknowledged. The National Institutes of Health (NIH) has submitted requests for proposals for PrEP implementation programs, including a call for “PrEP navigator resource development and dissemination” [76]. A search of the NIH U.S. National Library of Medicine reveals four clinical trials involving PrEP navigators [77] and the NIH Research Portfolio Online Reporting Tools (RePORT) reveals six projects on PrEP navigation for Black MSM, young Latino MSM, women upon release from incarceration, people who inject drugs (PWID), and methamphetamine users [78]. Though promising, PrEP navigation is not likely to address many of the barriers identified by this review—for example; at the Individual and Relationships Domains, primary care providers’ lack of knowledge in identifying PrEP candidates and prescribing PrEP; and, at the systems level, lack of funding and insurance, and stigma.

Therefore, the combination of and future testing of the effect of additional interventions is recommended. Clinic-based interventions should include trainings to assist both HIV-prevention and HIV-care providers in identifying appropriate PrEP candidates. Such training must target knowledge development (e.g., concerns about “real world” efficacy, toxicities, and future resistance); attitudes (e.g., prejudicial beliefs and assessments of the likelihood of risk behavior based on race or gender identity); and social norms about patients’ behavior (e.g., sexual risk and lack of adherence). Though provider training may improve PrEP implementation, system-level interventions (e.g., clinic funding and capacity) are needed to address population-specific barriers [79]. Navigation suggests help for patients in navigating healthcare systems to access insurance and co-pay assistance programs; nonetheless, system-level advocacy is also needed for expanding health insurance, coverage of medication, PrEP adherence counseling, and support services. Furthermore, interventions that directly address the effects of race- and gender-related stigma and racism may improve participation of underserved groups (e.g., Black MSM and transgender women) in the HIV Continuum of Care and thus their access to PrEP.

## Conclusion

Our approach and analysis highlight the structural dimensions of barriers to healthcare and public health and are consistent with literature addressing tensions between individual- and system-level barriers [80], structural stigma [81], and the shift from models of “cultural competency” to “structural competency” [82]. One possible limitation of our search terms is that we did not use the less common variation “preexposure prophylaxis,” and some papers using such term may have been missed. Nonetheless, based on our search, we identified, categorized, and analyzed barriers to PrEP implementation and interventions at the patient, provider, and healthcare-system levels. We argue for multilevel interventions that do not target providers, patients, or systems in isolation, but rather incorporate each of these levels into new models of implementation. We understand that our suggestion that interventions target all three areas is challenging, especially with regard to healthcare systems. As we know from current political discourse, healthcare systems are difficult to change and must be viewed in the context of larger political and structural realities that are challenging at best and nearly impossible to navigate at worst. An awareness of the challenges of healthcare systems, and the provision of concrete solutions for those needing and seeking PrEP, can be valuable means of improving healthcare-system interventions. Without attention to the ways structural factors affect individuals within healthcare systems, PrEP implementation may actually reinforce existing inequities that place the overwhelming burden of the HIV epidemic on more-vulnerable groups.
